# Data on the model of loneliness and smartphone use intensity as a mediator of self-control, emotion regulation, and spiritual meaningfulness in nomophobia

**DOI:** 10.1016/j.dib.2023.109479

**Published:** 2023-08-09

**Authors:** Triantoro Safaria, Nofrans Eka Saputra, Diana Putri Arini

**Affiliations:** Ahmad Dahlan University, Jambi University, Charitas Musi Catholic University, Indonesia

**Keywords:** Nomophobia, Emotion regulation, Self-control, Spiritual meaningfulness, Loneliness, Smartphone use intensity

## Abstract

The study was conducted in three locations: Yogyakarta, Palembang, and Jambi. A total of 355 psychology students from three different universities were recruited using purposive sampling. Among the participants, there were 313 females (88.03%) and 42 males (11.83%). The participants completed several questionnaires in the Indonesian version, including the nomophobia NMP-Q scale (Yildirim & Correia, 2015), the R-UCLA Loneliness Scale (Russell et al., 1980), the self-control scale (Tangney, Baumeister & Boone, 2004), the Difficulties in Emotion Regulation Scale (DERS; Gratz & Roemer, 2004), and the Spiritual Meaningfulness scale developed based on the theory of Pargament (2007).

Before commencing the analysis, the research team ensured the accuracy and reliability of the collected data sets. Participants who did not fully complete the questionnaire were removed from the sample. Ethical clearance for this study was obtained from the research ethics committee, and the researchers obtained permission from the respective university administrations for data collection. Prior to participation, all individuals agreed to take part in the study, provided voluntary informed consent, and were assured of the confidentiality and anonymity of their responses.

Specifications Table

**Table:** 

Subject	Psychology
Specific subject area	Psychology (General)
Type of data	Excel data file, Table
How the data were acquired	Questionnaires, we collected the data through paper-pencil surveys administered in the classrooms
Data format	Raw, Analyzed
Description of data collection	The data collection took place during June-July 2022, where a total of 355 participants completed the scales. The surveys were administered through offline paper-pencil surveys conducted in the classroom setting.
Data source location	The data for the research was collected from three distinct cities: Palembang, Jambi, and Yogyakarta. The study participants consisted of university students selected from Ahmad Dahlan University, Jambi University, and Charitas Musi Catholic University.
Data accessibility	Repository name: zenodo Direct URL to data: (https://doi.org/10.5281/zenodo.7933666)

## 1 Value of the Data

• The dataset provides some important information about nomophobia, emotion regulation, self-control, spiritual meaningfulness, loneliness, and smartphone use intensity in Indonesia.
• This data can be used in structural equation model (SEM), item response model, machine learning model, and other types of analysis.
• This data is taken from the cultural background of Indonesia, then it can be used for cross-cultural studies related to nomophobia.

## 2 Objective

The phenomenon of nomophobia has emerged as a relatively recent discovery within the past 10 years. Nomophobia, which stands for No Mobile-phone Phobia, has become recognized as a new mental health issue in the digital era [1,2]. It involves an irrational fear or anxiety that arises when individuals are unable to use, contact, communicate, or access their mobile phones. This fear encompasses concerns about missing out on information and feeling disconnected from virtual communication through the internet [3], [4], [5].

Previous studies have revealed the detrimental effects of nomophobia on individuals' psychological well-being. It has been significantly associated with problematic dependency, prohibited use, and excessive use of smartphones [6]. Additionally, it has been linked to obsessive behaviors [7,8], anxiety [9,10], panic disorders [11,12], stress [13], and the fear of missing out (FOMO) [14]. However, there is still much to be uncovered about nomophobia, and further research is needed to explore and understand the influencing factors, particularly among teenagers.

## 3 Data Description

We provide the research data in Excel file format, along with research questionnaires available in both English and Indonesian languages, accessible in the Zenodo repository. The questionnaire encompasses measurements of respondents' level of nomophobia, emotion regulation, self-control, spiritual meaningfulness, loneliness, smartphone use intensity, and demographic data (age and gender). This research was conducted in Yogyakarta, Palembang, and Jambi between June and July 2022. Purposive sampling was utilized to recruit 355 students from three universities in these cities to participate in the study. Out of the participants, 313 (88.2%) were female and 42 (11.8%) were male. It is worth noting that in Indonesia, the majority of psychology students are women, which results in an imbalance in the gender distribution within the sample data. Table 1 displays the descriptive statistics of all variables, while Table 2 presents the correlation matrix results between variables. Table 3 presents the demographic data of gender. Table 4 presents the age data of respondents.

**Table 1:** Descriptive statistic results of all variables.

Variables	N	Minimum	Maximum	Mean	SD
Nomophobia	355	14.00	52.00	35.8901	6.05
Self-control	355	12.00	43.00	30.6732	4.8
Smartphone use	355	4.00	16.00	10.4338	2.3
Emotion regulation	355	5.00	20.00	12.2085	2.5
Loneliness	355	11.00	40.00	20.2732	5.3
Spirituality meaningfulness	355	17.00	40.00	32.5183	4.9

**Table 2:** Correlation matrix between variables.

	1	2	3	4	5	6	7
Nomophobia	1.000	−0.211⁎⁎	.515**	−0.235**	.233**	.008	.047
Self-control	−0.211**	1.000	−0.296**	.434**	−0.454**	.466**	.124*
Smartphone use			1.000	−0.441	.201**	−0.085	−0.022
Emotion regulation				1.000	−0.249**	.149*	.109*
Loneliness					1.000	−0.249**	−0.004
Spirituality						1.000	.193**
Gender							1.000

⁎ *p* < .05.

⁎⁎ *p* < .01.

**Table 3:** Distribution of gender.

Gender	Frequency	Percent
Male	42	11.8
Female	313	88.2

**Tabel 4:** Distribution of respondents’ age.

Age	Frequency	Percent
20 years	98	27.6
21 years	62	17.5
22 years	59	16.6
23 years	21	5.9
24 years	3	.8
25 years	17	4.8
19 years	95	26.8

## 4 Experimental Design, Materials and Methods

### 4.1 Participants

The study was conducted in Yogyakarta, Palembang, and Jambi using purposive sampling to recruit 355 participants from three universities. These participants comprised 313 (88.03%) were female and 42 (11.83%) were male. Prior to their involvement, all respondents provided informed consent and willingly participated without any form of coercion. Moreover, the research team obtained permission from the respective schools and universities to collect data. As a gesture of appreciation, all participants received ballpoint pens upon completing the questionnaire. Data collection took place over a two-month period, officially commencing in June 2022 and concluding in July 2022.

### 4.2 Measurement

Nomophobia (MNPQ). Yildirim and Correia [15] developed the nomophobia (No Mo Phobia-Questionnaire) to evaluate the various dimensions of nomophobia. Factor Analysis (EFA) was used to identify four NMP-Q factors, including “unable to communicate (*I would feel nervous because I would not be able to receive text messages and calls*),” “lost connection (*I would be nervous because I would be disconnected from my online identity*),” “unable to access information and providing information (*I will feel uncomfortable without immediate access to information through my phone*),” and “giving up convenience (*If I could not check my smartphone for a while, I would feel a desire to check it*).” The varimax rotation approach employed principal component analysis (PCA) to test the correlation between these factors. In addition to these original factors, the questionnaire was further modified through the process of translating and adjusting item sentences. We utilized 13 items from the NMPQ questionnaire that had a total item correlation above 0.30. To adapt the scale for this study, a back-to-back translation process was conducted by two English-fluent experts. The resulting nomophobia scale comprised of four aspects: inability to communicate, losing connectedness, inability to access information, and giving up convenience. Participants responded to each item using a 4-point scale, choosing from the response options of Strongly Agree, Agree, Disagree, and Strongly Disagree. The Cronbach's alpha coefficient for the scale was 0.869 overall, with the total item correlation of the nomophobia scale ranging from 0.311 to 0.775.

*Loneliness*. To measure loneliness, we utilized the R-UCLA Loneliness Scale [16]. We selected 10 items from the R-UCLA questionnaire that had a total item correlation above 0.30, that evaluate emotional loneliness (*I feel rejected by my surroundings*) and social loneliness (*I don't have any close friends who are close to me*). To adapt the scale for this study, a back-to-back translation process was conducted by two English-fluent experts. Participants responded to each item using a 4-point scale, choosing from the response options of never, rarely, sometimes, and often. The loneliness scale had a total item correlation between 0.336 to 0.637 and an alpha-Cronbach coefficient of 0.722.

*Self-control.* The self-control scale, developed by Tangney, Baumeister & Boone [17], encompasses four dimensions: regulating thought and emotion (*I have difficulty concentrating*), resisting temptation (*I am good at resisting temptation*), breaking habits (*I find it hard to stop bad habits*), and maintaining good self-discipline (*People would say that I have strong self-discipline*). We utilized 12 items with a total item correlation above 0.30, that evaluate self-emotion appraisal, regulation of emotions, use of emotion, and others-emotion appraisal. During the adaptation process, a back-to-back translation was conducted by two English-fluent experts. Participants responded to each item using a 4-point scale, choosing from the response options of Strongly Agree, Agree, Disagree, and Strongly Disagree. The self-control scale exhibited a total item correlation ranging from 0.324 to 0.479, with an alpha-Cronbach coefficient of 0.614.

*Emotion regulation.* The Difficulties in Emotion Regulation Scale (DERS); Gratz & Roemer) [18] is utilized to assess emotion regulation. We selected 5 out of the 18 items that had a total item correlation above 0.30. This scale measures five aspects: strategies (*When I am angry, I struggle to finish other tasks*), non-acceptance (*When I am angry, I acknowledge my emotions*), awareness (*I have difficulty understanding my own feelings*), goals *(When I am angry, I have difficulty completing tasks*), and impulse (*When I'm upset, I lose control over my behaviors*). The adaptation process includes back-to-back translation, which is done by two English-fluent experts. Participants responded to each item using a 4-point scale, choosing from the response options of Strongly Agree, Agree, Disagree, and Strongly Disagree. The total item correlation of the emotion regulation scale ranges from 0.275 to 0.679. The alpha-Cronbach coefficient of this scale for measuring emotion regulation is 0.882.

*Spiritual Meaningfulness.* The spiritual level scale was created utilizing Pargament's theory [19], comprises eight items and was developed by the researchers. The researcher conducted the development of items for the spiritual meaningfulness scale through several stages, including defining the variable, reviewing existing literature, generating an initial item pool, evaluating the items, conducting pilot testing, reducing the number of items, refining the final items, and assessing the reliability and validity of the scale. Based on Pargament's theory, we formulated measuring items encompassing two aspects: theistic meaning (*I feel the presence of God the Creator*) and spiritual meaning (*When I worship, I feel enjoyment and peace*). Participants responded to each item using a 4-point scale, choosing from the response options of Strongly Agree, Agree, Disagree, and Strongly Disagree The total item correlation for the spirituality scale ranges from 0.311 to 0.769. The alpha-Cronbach coefficient for this scale is 0.842.

*Smartphone use.* To assess the level of smartphone usage among participants, a questionnaire on smartphone usage intensity was developed by the researchers. This questionnaire focused on two aspects: the frequency (*I use my phone every day*) and duration of smartphone usage (*I spend 12 h a day playing on my phone*). Participants responded to each item using a 4-point scale, choosing from the response options of Strongly Agree, Agree, Disagree, and Strongly Disagree. The total item correlation for the smartphone use scale ranged from 0.353 to 0.483. Furthermore, the alpha-Cronbach coefficient for this scale was determined to be 0.774.

As an additional clarification, all item scores in the Excel file have been scored according to their response scores. Other researchers can directly use the data without the need to reverse the scores. The research data can be downloaded from the Zenodo repository with the provided link in the data accessibility section. The following items on the self-control scale, namely KD1, KD2, KD3, KD4, KD5, KD7, KD8, and KD9, have been reverse-coded in the Excel data file. Additionally, the item RE5 on the emotion regulation scale has also been reverse-coded. Furthermore, the item KS9 on the loneliness scale has been reverse-coded. Lastly, the items SM6, SM7, and SM8 on the spiritual meaningfulness scale have been reverse-coded in the Excel data file.

## Ethics Statements

This study has received ethical clearance from the Research Ethics Committee of Universitas Ahmad Dahlan (Ethics Number: 012206071 KEP UAD). Additionally, the researchers have obtained a data collection permit from the university administration. All participants willingly agreed to take part in the study and provided their signature on a voluntary informed consent form. The confidentiality and anonymity of all participants were assured.

## CRediT authorship contribution statement

**Triantoro Safaria:** Conceptualization, Writing – original draft, Methodology, Data curation, Investigation, Writing – review & editing. **Nofrans Eka Saputra:** Data curation, Investigation. **Diana Putri Arini:** Data curation, Investigation, Writing – review & editing.

## Data Availability

Data on the Model of Loneliness and Smartphone Use Frequency as a Mediator of Self-Control, Emotion Regulation, and Spiritual Meaningfulness in Nomophobia (Original data) (zenodo).
